# Evaluating Country Performance After Transitioning From Gavi Assistance: An Applied Synthetic Control Analysis

**DOI:** 10.9745/GHSP-D-22-00536

**Published:** 2023-08-28

**Authors:** Robert John Kolesar, Rok Spruk, Tsheten Tsheten

**Affiliations:** a Palladium, Washington, DC, USA.; b Centre d’Études et de Recherche sur le Développement International, Université Clermont Auvergne, Clermont-Ferrand, France.; c School of Economics and Business, University of Ljubljana, Ljubljana, Slovenia.; d Royal Centre for Disease Control, Ministry of Health, Thimphu, Bhutan.; e National Centre for Epidemiology and Population Health, College of Health and Medicine, Australian National University, Australia.

## Abstract

**Introduction:**

Over the past decade, international development assistance for health has slowed. As donors seek to increase domestic cofinancing and ultimately transition countries from donor aid dependence, COVID-19 has severely constrained public budgets. The evaluation of sustainability and longer-term impacts of donor withdrawal is increasingly important. We assess vaccination coverage and post-neonatal mortality to estimate country performance of these outcomes among countries that no longer received assistance from Gavi, the Vaccine Alliance (Gavi) between 2000 and 2018.

**Methods:**

Using data from all countries receiving Gavi support between 2000 and 2020, we employed a synthetic control method to generate a pre-transition counterfactual with the same characteristics as the observation of interest to predict a future that empirically never existed. The synthetic unit is constructed from the weighted average of other units with good fit to the unit of interest before transition but did not transition.

**Results:**

We found substantial heterogeneity after transitioning from Gavi assistance. China, Guyana, and Turkmenistan overperformed their expected coverage rates; Albania, Bhutan, China, Guyana, and Turkmenistan maintained coverage over 90%; and Bosnia and Herzegovina and Ukraine reported precipitous drop-offs that fell well below their synthetic controls. We also observed a vaccination coverage decline in 2020 for several countries after transitioning and most synthetic controls, which we attribute to COVID-19-related service disruptions.

**Conclusions:**

We recommend that Gavi adjust its transition model to systematically assess contextual externalities and risk. In addition, countries that no longer receive Gavi assistance can leverage technical assistance and communities of practice to mutually assist each other and other countries advancing toward transition. This could also foster intracountry accountability after transition. We also recommend that Gavi systematize post-transition assessments and evaluations that leverage the expertise and experience of graduated countries to encourage cross-learning.

## Abstract (precis)

After transitioning from Gavi support, most countries in this analysis maintained or improved key outcomes compared to expected performance. Transition planning should include assessing risk factors and engaging country actors for post-transition assistance.

## Teaser

**Key Findings:**

- Despite context-specific differences in vaccination coverage among countries after transitioning from Gavi assistance, most countries successfully maintained or further improved key outcomes compared to their expected performance.
- Unstable or decreasing vaccination coverage before becoming fully self-financing may be considered risk indicators, particularly among countries facing governance challenges.
- Annual fluctuations in vaccination coverage may have a limited impact on the overall trajectory of post-neonatal mortality when the rate is relatively low.

**Key Implications:**

- Synthetic control, a quasi-experimental method, can be used to evaluate large-scale global health program transitions. The results can inform approaches to safeguard and further gains achieved with external financial assistance, thereby maximizing the potential for success.
- International donors should assess contextual externalities and risks to potentially slow down the process of transitioning countries from assistance when needed.
- Country actors can leverage mechanisms for post-transition technical assistance and accountability and provide guidance to transitioning countries.
- Gavi can systematize post-transition assessments and evaluations that engage the expertise and experience of countries that no longer receive its support.

## BACKGROUND

Childhood immunization is among the most cost-effective and long-lasting health interventions.^1^^,^^2^ In addition to the direct impacts on the Sustainable Development Goal 3 of good health and well-being, immunization contributes to 14 of the 17 goals.^3^^,^^4^ Established in 2000, Gavi, the Vaccine Alliance (Gavi) has disbursed more than US$18 billion (excluding COVID-19 funds) to improve equitable access to vaccines and improve health and well-being, primarily among children in the poorest countries.^5^^,^^6^ There is strong evidence showing that Gavi assistance has increased immunization coverage and reduced child mortality.^7^^–^^10^

Over the past decade, the slowing growth of international donor assistance for health has elevated the discussion about countries’ self-reliance and the transition from donor aid dependence.^11^^–^^13^ The Immunization Agenda 2030 calls out country ownership as key to this transition because the most important actions will be the responsibility of individual countries, with domestic financing remaining the most important contribution to immunization.^14^ Undoubtedly, governments need to do more to ensure robust funding in a sustainable and predictable manner.^15^ However, the post-COVID-19 world is expected to face exacerbated, competing health needs and constrained economic growth.^16^ As donors seek to increase domestic cofinancing and ultimately transition countries from external support, COVID-19 places enormous pressure on public budgets.^17^ Economic growth slowed among many low- and lower-middle-income countries, increasing the likelihood of fiscal austerity measures (i.e., reduced government spending).^16^ The International Monetary Fund recently noted that an increase in interest rates from the U.S. Federal Reserve would likely have severe impacts on emerging economies. To address their rising debt and inflation, several low- and middle-income countries have started to adjust monetary policy and are preparing to reduce spending.^18^

It is important to note that an exclusive focus on replacing external assistance with domestic financing for a specific program is problematic because it limits the sustainability issue to revenues and the scope for action to the specific, externally financed health program.^19^ The successful transition from large donor programs to country self-reliance faces many commitment and capacity challenges related to leadership, financing, programming, and service delivery.^20^ There is a clear need for early planning and monitoring of the transition along these domains. More specific challenges include: (1) limited economic growth and political will of governments to replace donor-funded programs; (2) limited coordination function of governments and weak decision-making power of coordinating mechanisms obscuring the latter’s future role; and (3) lack of health program maturity, such as inadequate function of national procurement and supply chain management systems, which can interrupt the supply of quality-assured commodities.^12^^,^^15^^,^^21^

The successful transition from large donor programs to country self-reliance faces many challenges related to leadership, financing, programming, and service delivery.

**FIGURE 1:**
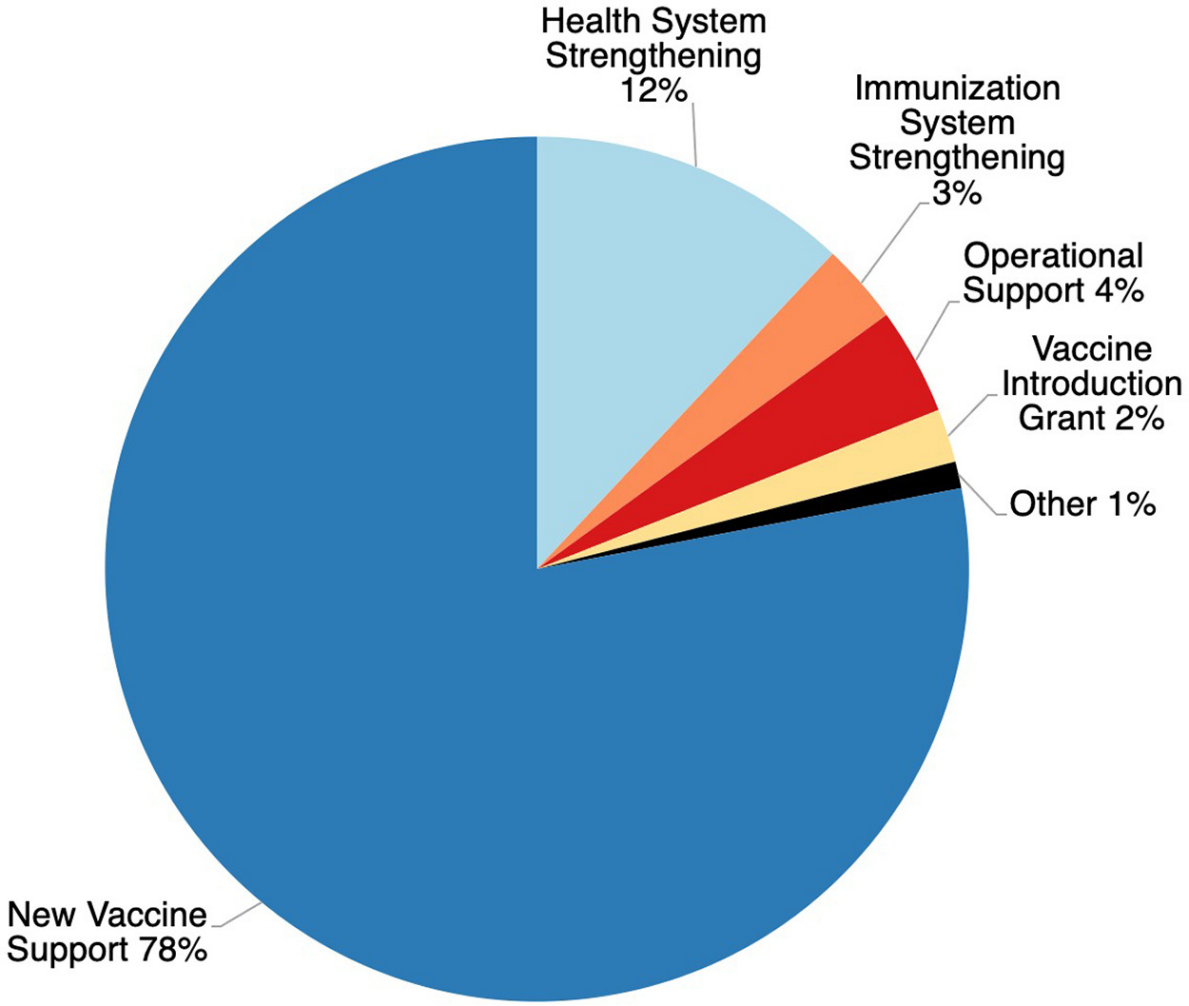
Proportional Breakdown of Gavi Financing by Major Program Category^a^ ^a^ Authors’ calculations based on Gavi financing data.^22^

Figure 1 illustrates the proportion of country support provided by major program categories.^22^ Fifteen percent of all country support was dedicated to health system strengthening (12%) and immunization system strengthening (3%). This represents a US$2.7 billion investment in systems strengthening. Other support (1%) includes financing for civil society organizations, product switch grants, graduation grants, and cold chain equipment optimization.

**FIGURE 2:**
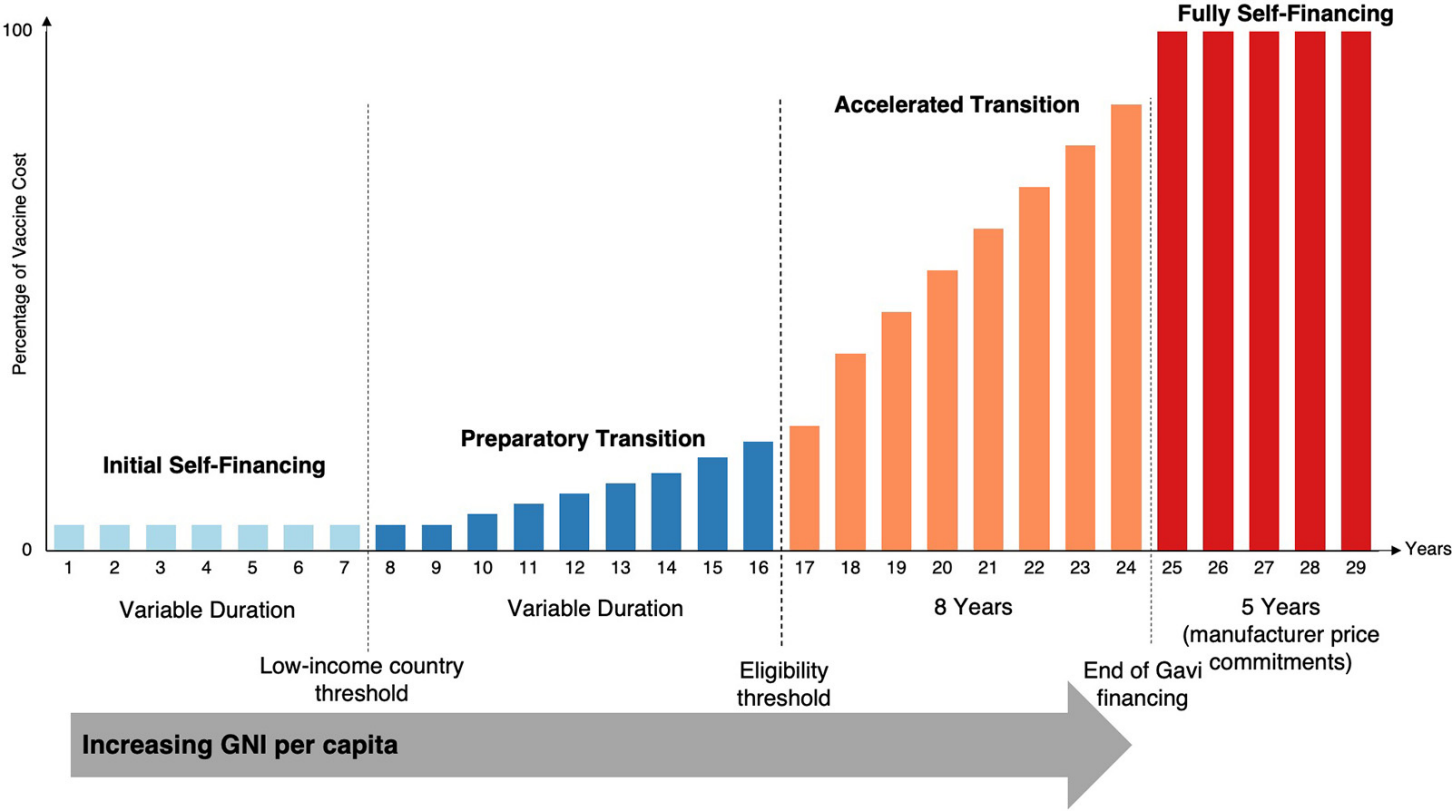
The Gavi Model: Country Contributions to Vaccine Costs^24^^,^^29^^,^^30^

To promote the transition to financial self-reliance and sustainability, Gavi has increasingly invested in health system strengthening and transition planning, including the progressive cofinancing or co-procurement of vaccines introduced with Gavi support.^23^^–^^26^ Initiated in 2008, Gavi’s transitional assistance model and cofinancing policy have evolved over time (Supplement 1). Gavi’s current policy, adopted in 2015 (and updated in 2016, 2018, and 2023), bases country eligibility on gross national income per capita less than US$1,085 in 2023 (Box).^27^ These countries, typically low-income and called “initial” countries, are eligible to apply for new vaccine and health system support. Once gross national income per capita increases above this threshold, a country becomes a phase 1 or preparatory transition country and enters into the cofinancing mechanism. With gradually increasing levels of self-financing, countries move on to phase 2 or the accelerated transition phase, when Gavi is actively reducing its support for vaccine and health system support.^24^^,^^28^ Effective January 2023, Gavi extended the accelerated transition phase from 5 to 8 years.^29^ The Gavi transitional assistance model is illustrated in Figure 2.^24^^,^^29^^,^^30^

BOX Country Perspective on Transition From Gavi Assistance

Gavi’s current transition framework focuses on gross national income per capita thresholds. It is important to note that the World Bank classifies economies into income country groups for analytical purposes.

Moreover, the fact that a country has passed a threshold does not necessarily change the underlying country situation in the immediate or short term. A country’s economic growth, capacity, and commitment evolve incrementally, and progress is not always linear.

Although Gavi’s updated “accelerated transition eligibility threshold” also requires a country to cofinance at least 35% of vaccine costs, this approach does not consider the broader context and health system performance, nor does it necessarily capture a country’s preparedness in terms of overall financing and institutional capacity.

The evaluation of sustainability and longer-term impacts of donor withdrawal has become increasingly important to ensure that countries sustain or further advance their health development goals.^31^^,^^32^ Bao et al. assert that “monitoring and evaluating large-scale global health program transitions can strengthen accountability, facilitate stakeholder engagement, and promote learning about the transition process and how best to manage.”^20^ However, the literature focuses on commitment and capacity challenges, which leaves a gap in comparative analyses on system performance (e.g., maintaining services and health gains) in transitioning countries, particularly those countries that have transitioned from Gavi support.^33^ This study evaluates and compares vaccination coverage and post-neonatal mortality to estimate country performance for these outcomes among the 8 countries that transitioned from Gavi assistance between 2000 and 2018.

## DATA AND METHODS

The data set was constructed based on the 76 countries receiving Gavi assistance between 2000 and 2020. We used the full range of available data (i.e., starting in 2000), maximizing the pre-graduation period to construct each country’s synthetic control. We limited the analysis to 2020 due to incomplete data beginning in 2021. The outcomes of interest were diphtheria, tetanus toxoid, and pertussis third dose (DTP3) coverage among children aged 1 year, measles first dose coverage among children aged 1 year, and post-neonatal child mortality. Data for these variables were sourced from the World Health Organization.^34^ DTP3 coverage and measles by age 1 year are considered good indicators of immunization program performance.^35^ In addition, vaccine-preventable infectious diseases account for about 13% of all child mortality.^36^ We focused on post-neonatal child mortality (i.e., children aged 1–59 months) as children do not receive 3 doses of DTP nor the first dose of measles vaccination in the first month of life.

A range of potential matching variables were considered, including those relating to specific transition challenges identified from the literature and previously noted. However, matching variables were limited to those for which we could identify standard indicators with data for the included countries over the time period. Matching variables included per capita Gavi disbursements and non-Gavi health development assistance, population, fertility, government effectiveness, political stability, and corruption control, as well as country characteristics including land area, location (i.e., coordinates, coastal proximity, and ocean access), roadways, and topographic/climate type. These publicly available data were sourced from Gavi, the Institute for Health Metrics and Evaluation, the World Bank, the World Health Organization, and published literature.^22^^,^^37^^–^^40^ Data gaps were assessed and addressed either by multiple imputation, interpolation, or Wikipedia searches (in the case of country characteristics).

Countries were considered transitioned in the year of a Gavi “graduation grant” and/or if the country received less than US$100,000 in Gavi support. Sri Lanka received a graduation grant in 2015 but received US$1,786,323 in 2017 and US$313,930 in 2018. Both Armenia and Timor-Leste received graduation grants in 2017 but received US$201,692 and US$132,070 in 2018, respectively. Moldova received a graduation grant in 2016 but received US$272,242 in 2017 and US$272,288 in 2018. Likewise, Honduras received a graduation grant in 2015 but received US$3,338,026, US$287,042, and US$1,544,685 in 2016, 2017, and 2018, respectively. Therefore, these countries were not considered as fully self-financing regarding Gavi financial assistance. Like Armenia and Timor-Leste, Azerbaijan and the People’s Democratic Republic of Laos received Gavi support in 2018 and were not assessed because the post-transition period was limited to 2 years. The 8 countries that met the transition inclusion criteria and assessed in this study are Albania (2014), Bhutan (2015), Bosnia and Herzegovina (2011), China (2005), Georgia (2017), Guyana (2017), Turkmenistan (2005), and Ukraine (2008). Summary statistics are presented in Table 1.

**TABLE 1.** Summary Statistics of Outcome Variables for Evaluating the Performance of 8 Countries After Transitioning From Gavi Assistance

	**Mean**	**Median**	**SD**	**Minimum**	**Maximum**
DTP3 coverage %	78.11	83.00	17.66	19.00	99.00
Measles coverage, %	76.90	80.00	17.60	16.00	99.00
Post-neonatal mortality rate	48.23	40.41	34.62	1.63	181.93
Matching variables					
Development assistance for health net Gavi	1.401e+08	59,037,880	1.974e+08	0	1.223e+09
Gavi disbursement	10,328,144	2,512,107	22,065,331	0	1.722e+08
Population	43,630,501	11,353,140	1.507e+08	84,405	1.380e+09
Fertility rate	4.24	4.44	1.54	1.22	7.68
Political stability score	−0.71	−0.58	0.85	−3.31	1.42
Government effectiveness score	−0.85	−0.79	0.49	−2.45	0.39
Corruption control score	−0.79	−0.81	0.46	−1.87	0.76
Land area (1,000 km)	48,920	22,754	61,299	81	297,319
Latitude	10.84	10.44	17.51	−29.58	47.21
Longitude	32.82	29.92	52.35	−86.6	159.6
Tropical climate	54.86	64.88	42.25	0	100
Coastal proximity (1,000 km)	32.12	13.16	36.30	0	100
Island	0.14	0	0.35	0	1
Landlocked	0.29	0	0.45	0	1
Roads (1,000 km)	27.15	13.50	38.60	1.00	209.64

Abbreviations: DTP3, diphtheria, tetanus toxoid, and pertussis third dose; SD, standard deviation.

Policy assessments increasingly pose methodological challenges for impact evaluation of large-scale health initiatives in low- and middle-income countries.^41^ Synthetic control is a nonparametric, data-driven procedure that uses a latent factor model to generate a counterfactual with the same characteristics as the observation of interest (before transitioning from Gavi assistance) to predict a future that empirically never existed.^42^ The counterfactual or synthetic unit is constructed from the weighted average of other units that most closely resembles (i.e., good pre-treatment fit to) the actual unit of interest before treatment or exposure but were not exposed to the treatment/intervention.^43^^,^^44^ The evolution of the outcomes for the resulting synthetic control is an estimate of the counterfactual of what would have been observed for the country of interest if Gavi assistance had continued.^42^^,^^45^ This study applies the generalized synthetic control method to enable the calculation of uniform *P* values for the full sample placebo estimates.^46^

We considered other possible quasi-experimental methods. For example, difference-in-differences can be used to assess the differential effect of exposure on both treated and control groups. However, this method is generally applied to situations with a relatively large number of exposed units. Moreover, effect differences among exposed units would be masked by grouping. To the best of our knowledge, the methods used for the evaluation of Gavi transition thus far are not similar to this present study.^7^^–^^10^

Synthetic control has been used to assess governance on progress toward universal health coverage and health outcomes, as well as to estimate the impact of donor programs on child mortality in low- and middle-income countries.^47^^–^^49^ We computed the statistical significance of the counterfactual public health outcomes in the hypothetical absence of the phased transition from Gavi assistance by estimating the same outcome-linked model specification on each unaffected country and obtained the distribution of placebo effects by iteratively shifting the treated country in the donor pool. Supplement 2 provides a more detailed technical methodological description. All analysis was completed using Stata version 17 SE.

## RESULTS

**FIGURE 3:**
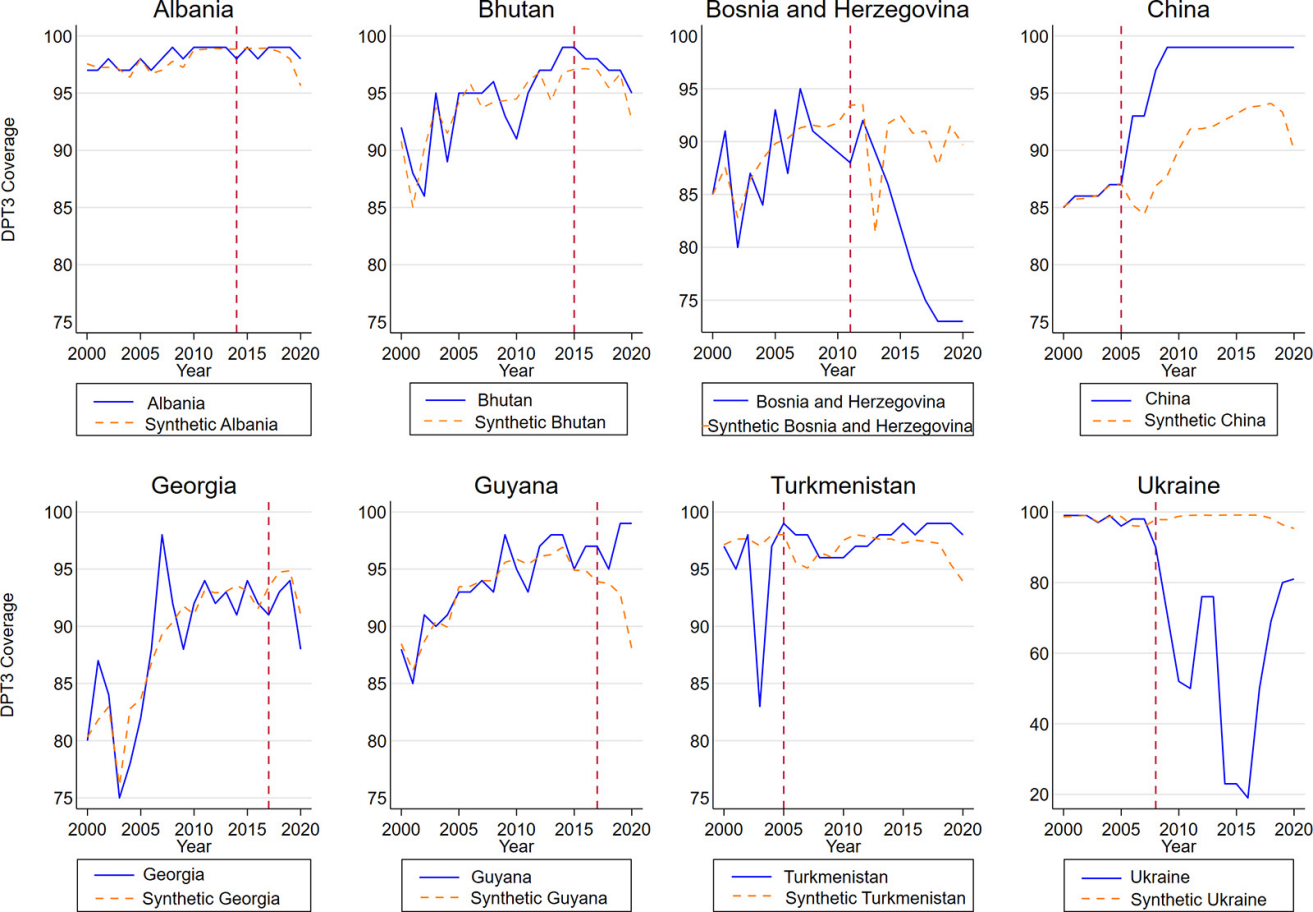
Diphtheria, Tetanus Toxoid, and Pertussis Third Dose Coverage and Synthetic Controls Among 8 Countries Before and After Transition From Gavi Assistance, 2000–2020

We present a summary of analytical results; Supplement 3 includes a detailed results description. Figure 3 shows the observed trajectories and synthetic counterparts of each country’s DTP3 coverage from 2000 to 2020 among the 8 countries that transitioned from Gavi support. As noted by the World Bank, immunization coverage levels are the result of a health system’s inputs and efforts; therefore, they frequently vary from year to year in each country.^50^ This rendered some variation between the treated countries and their synthetic peers in the pre-transition period. The results reveal heterogeneity in the DTP3 coverage trend after transition, corroborating the notion that the effect of transition was far from uniform. At the end of 2020, Albania, Bhutan, Guyana, and Turkmenistan overperformed their synthetic controls. Most notably, China achieved (near) universal DTP3 coverage by 2010, and in that case, the post-transition period exhibited the characteristics of a rapid and sustained departure from its synthetic control.

The results reveal heterogeneity in the DTP3 coverage trend after transition, corroborating the notion that the effect of transition was far from uniform.

By contrast, the estimates also revealed a negative effect in Bosnia and Herzegovina and Ukraine. Synthetic Bosnia and Herzegovina maintained coverage of more than 85% during the post-graduation period (exempting 2013). In relation to Ukraine, we observed a rampant deterioration in coverage post-transition until 2015 followed by a rapid recovery until 2020, when Ukraine’s DTP3 coverage lagged its synthetic control. Notably, synthetic Ukraine maintained coverage of more than 90% during the entire post-graduation period. Figure 3 also highlights that Bosnia and Herzegovina, Turkmenistan, and Ukraine had important DTP3 coverage instability or deterioration before support from Gavi ended.

**FIGURE 4:**
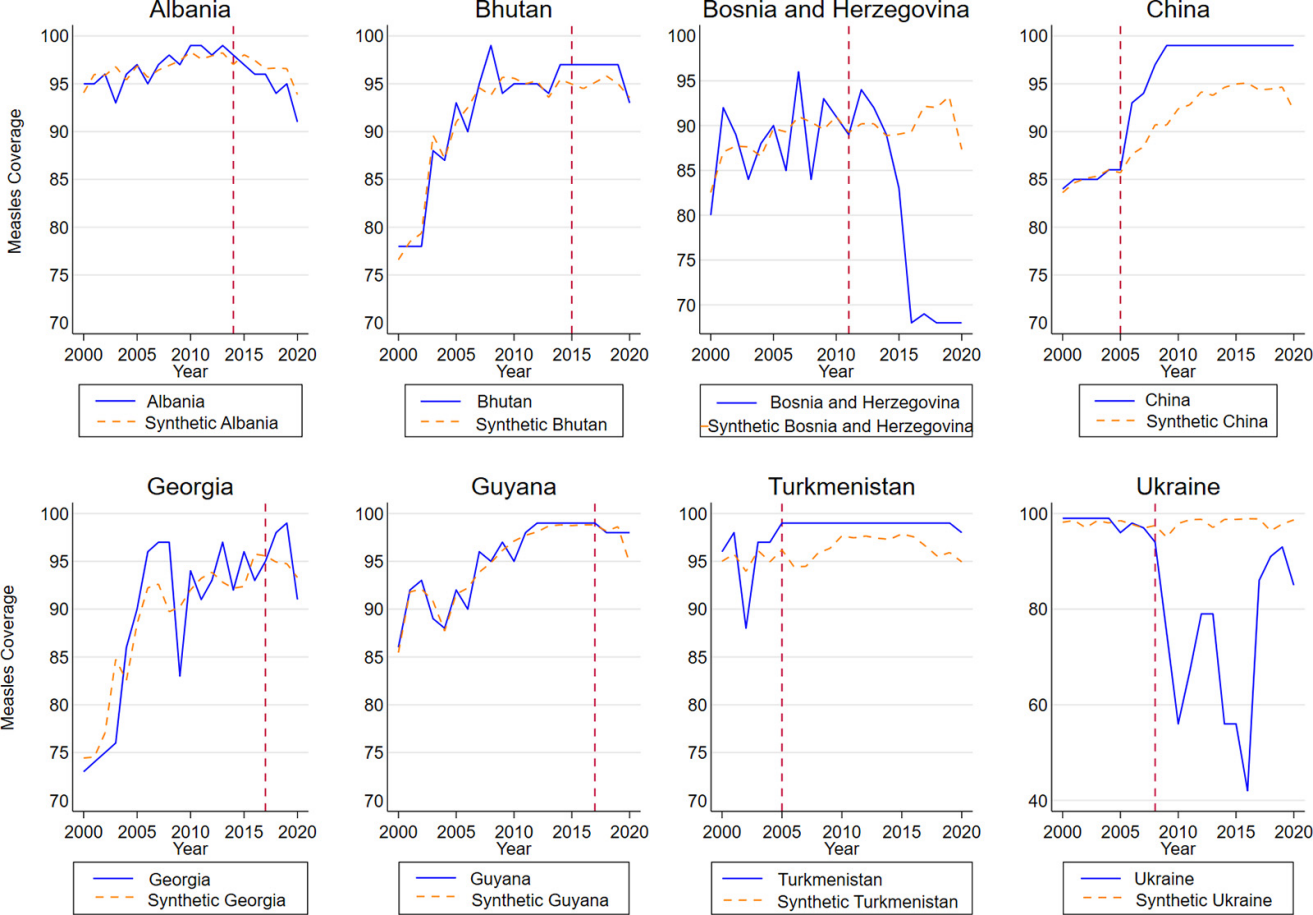
Measles Coverage and Synthetic Controls Among 8 Countries Before and After Transition From Gavi Assistance, 2000–2020

Figure 4 illustrates the observed trajectories of each country’s measles coverage and their respective synthetic control estimates. The results are very similar to those shown in Figure 3. We observe a general trend of coverage deterioration in Albania after graduation. However, this trend is closely aligned with its synthetic control. In addition, after graduation from Gavi support, Bhutan and Georgia outperformed their respective synthetic controls, with both experiencing a coverage deterioration. China and Turkmenistan consistently outperformed their synthetic controls with sustained (near) universal coverage. This is in stark contrast to all other graduated countries. Guyana maintained high coverage after graduation, outperforming its synthetic control.

Finally, before graduation, we observed unstable measles coverage in Bosnia and Herzegovina and decreasing coverage in Ukraine. In relation to the former, the observed data show a sustained deterioration of measles vaccination coverage after Gavi graduation. In the final year of Gavi support, measles coverage stood at 90%, like its synthetic control. By the end of the sample period in Bosnia and Herzegovina, the vaccination rate plummeted to 68%, lagging its synthetic counterpart by about 25 percentage points (95% confidence interval=−23.1, −26.9). In relation to Ukraine, the observed measles vaccination rate tended to decrease substantially in the first years after graduation until 2016, followed by a rapid and uninterrupted recovery. Ukraine’s rate of vaccination against measles in 2020 was around 12 percentage points (95% confidence interval=−5.5, −18.5) lower relative to its synthetic control.

**FIGURE 5:**
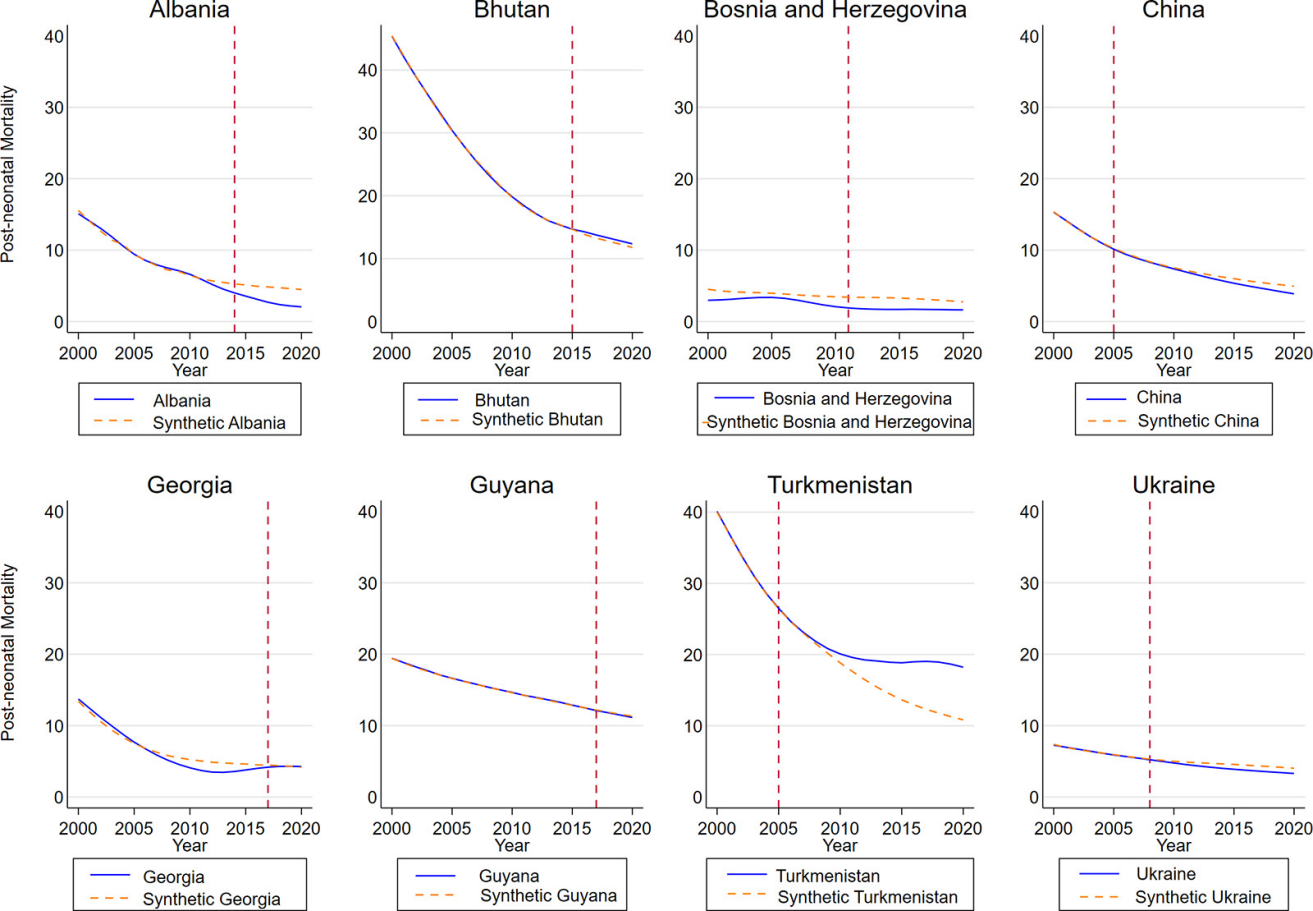
Post-Neonatal Mortality Rates and Synthetic Controls Among 8 Countries Before and After Transition From Gavi Assistance, 2000–2020

Figure 5 shows post-neonatal mortality rates and the respective synthetic control estimates for the 8 countries. In contrast to immunization coverage indicators, which frequently vary from year to year in each country, child mortality responds to a number of determinants and changes slowly.^50^ Thus, the synthetic control estimator provides an excellent fit between the treated countries’ trajectories and their synthetic control groups with almost zero imbalance therein.

**FIGURE 6:**
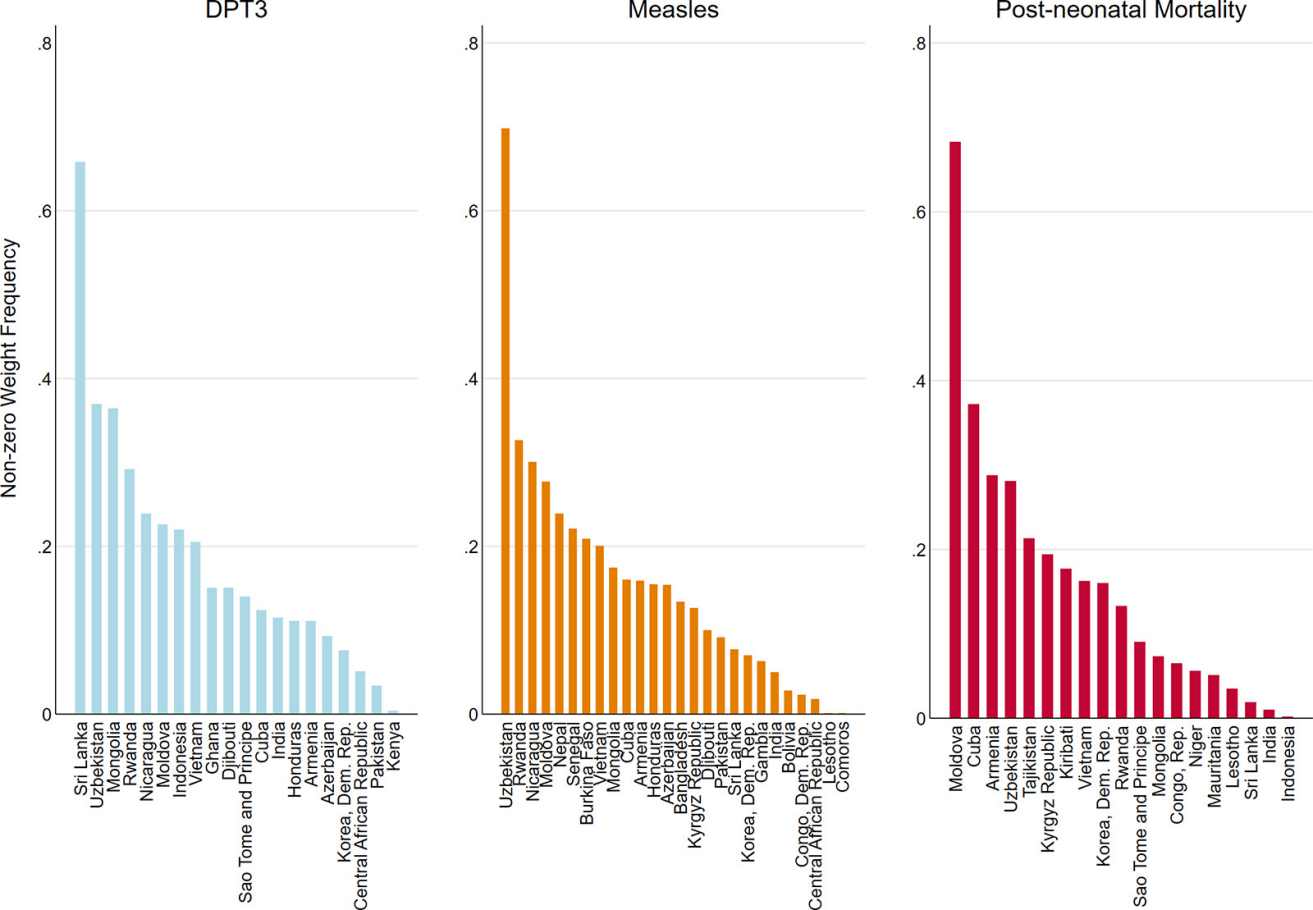
Condensed Composition of Synthetic Control Groups

As previously described, (1) the donor pool included all countries that had not yet transitioned from Gavi support through 2020; (2) each country that transitioned from Gavi assistance is considered as treated; and (3) the synthetic unit was constructed from the weighted average of other units with good pre-treatment fit to the unit of interest before transition but did not transition. Figure 6 reports the condensed composition of synthetic control groups, summarizing the frequency of non-zero weight for each donor country not affected by the treatment itself. A higher frequency indicates a stronger and more influential contribution of that donor country to the vaccination and mortality trajectories. This is because it most closely matches and reproduces the trajectories of the treated countries’ vaccination coverage and mortality paths in the period before Gavi assistance ended.

We computed the statistical significance of the counterfactual public health outcomes and obtained the distribution of placebo effects. The resultant *P* values can be interpreted as “the probability of obtaining an estimate at least as large as the one obtained for the unit representing the case of interest when the intervention is reassigned at random in the data set.”^51^ Table 2 presents the key parameters from this placebo analysis. The results suggest that assigning the transition date at random to the countries that did not transition does not yield statistically significant estimates, providing reasonable plausibility of our estimates.

**TABLE 2.** Full-Sample Placebo Estimates for Average Treatment Effect on the Treated

	**Placebo ATT Coefficient**	**Standard** **Deviation**	**95% Confidence Interval**	**Placebo *P* Value**
DTP3 coverage	0.429	2.126	−2.632, 6.234	.84
Measles coverage	0.975	2.484	−2.683, 6.125	.69
Post-neonatal mortality rate	−0.080	0.830	−1.452, 1.895	.92

Abbreviations: ATT, average treatment effect on the treated; DTP3, third dose of diphtheria, tetanus toxoid, and pertussis vaccine.

We also completed a more rigorous in-time placebo analysis by deliberately assigning the transition from Gavi to the wrong policy year (i.e., 5 years before the actual transition year) for all treated countries and outcomes under investigation. The findings suggest no effect of the quasi-intervention and reiterate the results from our prior analysis.

In addition, we completed a leave-1-out sensitivity analysis, and it did not yield any change in the results. We attribute this to the fact that: (1) all non-transitioned countries were present in the donor pool, and (2) we did not observe specific countries from the donor pool with a massive weight loading.

Finally, we computed the *P* values on the null hypothesis behind the treatment effect through the permutation of the Gavi transition to the unaffected countries by undertaking an in-space placebo analysis similar to Abadie et al.^42^ The underlying *P* values were computed through the comparison of the root mean square error before and after the transition between the graduating countries and their peers in the donor pool to which the treatment is reassigned. It should be noted that the *P* values reflect the degree of statistical significance behind the estimated treatment effect. High values indicate little or no significant effect of transition, whereas low values indicate stronger support for the notion of significant change in key public health outcomes after Gavi transition. In Supplement 4, we report the full set of country-year-indicator *P* values.

The evidence suggests several noteworthy insights from our analysis. In particular, we found statistically significant improvement in DTP3 vaccine coverage after transitioning from Gavi assistance in Albania, China, and Guyana. In the 3 respective cases, the *P* values on the null hypothesis gradually approached the 10% threshold by 2020. As expected, the evidence from China indicated no reduction in the *P* value once the universal coverage was achieved by 2010. By contrast, the transition from Gavi was associated with a statistically significant reduction in the DTP3 vaccine coverage in Bosnia and Herzegovina and Turkmenistan. The computed *P* values fell within 10% significance bound and remained stable up until the end of the period after transitioning from Gavi support, indicating a sustained reduction instead of a temporary reduction in vaccine coverage. In the measles placebo simulations, we found evidence of significant improvement in vaccine coverage in Turkmenistan as opposed to a sustained reduction in vaccine coverage in Bosnia and Herzegovina, temporary reduction in Ukraine, and a transition to universal vaccine coverage in China. Lastly, the placebo evidence also indicated a relatively large increase in the post-neonatal mortality rate in Turkmenistan after Gavi transition compared to its synthetic control. The computed *P* values were strikingly low and stable throughout the entire post-treatment period and appeared within the 10% bound. There was no evidence of statistically significant increases in post-neonatal mortality among the other 7 countries.

## DISCUSSION

This study assesses country performance after transitioning from Gavi assistance with a focus on 3 key outcome variables: DTP3 and measles coverage by age 1 year are considered good indicators of immunization program performance^35^; likewise, vaccine-preventable infectious diseases account for about 13% of all child mortality.^36^ Countries demonstrated substantial heterogeneity of key public health indicator trends after Gavi graduation. China, Guyana, and Turkmenistan sustained higher than expected vaccination coverage compared to their respective synthetic controls, suggesting a very successful transition. Albania maintained its DTP3 coverage, outperforming its synthetic control. However, Albania’s measles coverage indicated a gradual deterioration, similar to what was expected as estimated by its synthetic control. Bhutan and Georgia demonstrated notable coverage variation and deterioration, although this was consistent with their respective synthetic controls. Overall, post-transition performance of Albania, Bhutan, and Georgia can be characterized as meeting expectations.

Countries demonstrated substantial heterogeneity of key public health indicator trends after Gavi graduation.

Exempting Ukraine, we observed a sharp decline in DTP3 and measles coverage among all synthetic controls in 2020 and attribute this to COVID-19-related service disruptions among matched countries. There was a similar decline among 5 countries: Albania, Bhutan, Georgia, Turkmenistan, and Ukraine (measles only). Notably, Bosnia and Herzegovina, China, Guyana, and Ukraine (DTP3 only) sustained 2019 coverage for 2020.

Bosnia and Herzegovina and Ukraine were characterized by rampant coverage decline and underperformance compared with pre-transition levels and their synthetic controls. Importantly, Ukraine demonstrated some recovery by 2020. The observed decline in Bosnia and Herzegovina’s immunization rates have been attributed to growing vaccine hesitancy, misinformation in social media, lack of trust in the health system, a shortage of health workers, and supply issues.^52^ A recent study in Bosnia and Herzegovina on health worker vaccination practices found the use of false contraindications to postpone vaccination and poor skills in tailoring communication with parents, lack of implementation of mandatory vaccination, no uniform recall and reminder system or system for detecting under-vaccinated children, staff shortages and lack of time to discuss vaccination with parents, and tendency to blame external factors (e.g., anti-vax movement and a fear of being blamed for adverse events).^53^

A dramatic decline in Ukraine’s vaccination coverage began in 2007, which coincided with the Ukrainian political crisis. In addition, widespread rumors of side effects were attributed to corruption and low-quality vaccines procured by the Ministry of Health from allegedly unqualified manufacturers.^54^ Vaccinations, purchased in large volumes, make them highly vulnerable to corruption risks.^55^ Public mistrust was triggered by Internet disinformation that falsely linked measles vaccination to the death of a boy aged 16 years.^56^ This was compounded by institutional challenges, including lack of financing, distribution weaknesses, and the abandonment of school vaccine requirements. These factors explain the significant coverage drop that coincided with the end of Gavi support.^57^

Three key insights can be drawn from our analysis of the respective mortality impact after transition. First, a discernible decrease in the mortality rate can only be expected in those countries that had a notably higher mortality rate relative to the benchmark levels. The results revealed a sustained drop in the post-neonatal mortality trajectory in Albania. By contrast, the trend decrease in post-neonatal mortality in Turkmenistan underperformed, with reductions leveling off in 2012. Thus, we found that all transitioned countries, except Turkmenistan, either outperformed or matched their respective synthetic controls in relation to post-neonatal mortality. We could not find any supplemental information in the literature to explain the plateauing of post-neonatal mortality in Turkmenistan.

We found that all transitioned countries, except Turkmenistan, either outperformed or matched their respective synthetic controls in relation to post-neonatal mortality.

In addition, we note that Albania, Bosnia and Herzegovina, China, Georgia, and Ukraine had very low post-neonatal mortality rates at transition, with near exact pre-transition matching among their respective synthetic counterparts. Because these countries already had relatively low mortality rates, the potential for additional improvement was limited. Given the temporal variability in vaccination coverage among the 8 countries, this suggests that fluctuations in vaccination coverage have a limited impact on the post-neonatal mortality rate when the rate is relatively low. Finally, the post-transition non-discrepancy between the transitioned countries and their control groups suggests that those countries maintained progress in post-neonatal mortality rate reductions similar to what would have been expected if Gavi support had continued.

In summary, some countries such as Albania, Bhutan, Guyana, and Ukraine had consistently higher vaccination rates than their peers at the pre-transition stage. The effect of exit from Gavi’s support on the vaccination rates was either both negative and large in Bosnia and Herzegovina or large, negative, and temporary in Ukraine. We also found some evidence of a small but progressive reduction in post-neonatal mortality apart from Turkmenistan, which underperformed when compared to its synthetic control. The interpretation of our estimates is consistent with earlier studies. For instance, a multicountry study (including Bhutan and Georgia) found that country-level capacity to assume and hold responsibility for their immunization programs was highly heterogeneous.^11^ In addition, evidence indicated that a multitude of factors impacted a country’s ability to transition off Gavi assistance. Examining the sources of inefficiency and sustainability of Gavi’s Health System Strengthening grants, Mimche identified health workforce weaknesses, poor governance, an excessively long implementation period, and disrupted focus toward the procurements and service provision as factors that exacerbated weak plans to exit from support.^58^ A prior study highlighted potential complicating externalities that can impact graduation, such as large-scale external shocks, newly evolving pandemics, and fragile domestic institutional environments.^15^

We note several examples of additional Gavi financing after graduation grants (refer to the Data and Methods), which effectively slowed down the transition process. This suggests that Gavi took a cautious and flexible approach to transition, likely in situations determined as high risk. However, the unstable or decreasing vaccination coverage in Bosnia and Herzegovina and Ukraine in the year(s) before graduation could have been considered risk indicators and could have been considered a reassessment trigger. Thus, we recommend that Gavi systematically assess contextual externalities and risk to slow the process when needed and note Gavi’s extended accelerated transition time frame from 5 to 8 years.^29^

Essential elements to improve country ownership and support countries to better prepare for transition beyond exogenous factors include: (1) updated national policies that reflect current national priorities; (2) increased collaboration between the ministries of finance and health to develop and adopt a viable vaccine financing policy; (3) adoption of legislation that prioritizes financing of vaccines and immunization; (4) a strong national logistics system; (5) increased reliability of resources for immunization; and (6) leveraging of health financing reforms.^59^^,^^60^ Bao et al. assert that a “sustainable transition requires a clearly articulated vision of long-term impact, explicit and transparent transition policies, clear time frames for transition, donor coordination, and evaluation of long-term impacts of donor withdrawal.”^20^

### Limitations

There are several important limitations and caveats related to both ex-ante and ex-post components of this analysis. First, the analysis is constrained by the time span due to ex-ante data limitations that do not permit a longer pre-transition period. However, as Gavi was established in 2000, we believe it is a logical base year.

Second, this analysis does not present an average post-transition treatment effect among the 8 countries. This is because doing so would mask heterogeneity, which is an important finding. Also related, this analysis does not provide causal identification nor exhaustive evidence for the post-transition effects. As the findings indicate important differences among the 8 countries, we believe that the context and externalities are highly country specific.

Third, the observational nature of our analysis uses individual country-level data to examine the evolution of vaccination coverage and post-neonatal mortality to assess selected outcomes at the national level after Gavi transition. The benefit of this quasi-experimental approach is that it enables the estimation of the counterfactual. Although an analysis using household survey data would clearly provide more meaningful insights into the post-transition effect within a given country, the primary focus of this study was to assess post-transition country performance. Moreover, such multiyear data was not available across the graduating countries.

Fourth, this analysis does not investigate national inequities nor subnational effect disparities. This would be particularly useful for large countries with nontrivial economic, health-related, and demographic differences within their territories. For instance, a large-scale positive effect of the immunization program on the DTP3 and measles vaccination coverage in a particular country likely differs across wealth quintiles, ethnicities, religious groups, residences (i.e., rural/urban), provinces, and regions. The interpretative side of the analysis may be acutely difficult without any further knowledge of the distribution of outcomes across these dimensions. A more nuanced and spatially disaggregated analysis could exploit additional angles and dimensions of the immunization coverage to further uncover policy-relevant issues.

Fifth, this analysis does not fully address the external validity concerns that lie in the spectrum of policy-relevant considerations. Although Gavi has been generally praised for its innovativeness, effectiveness, and less bureaucratic approach compared to other international development assistance entities, addressing the external validity would necessitate the existence of a similar immunization financing mechanism that benefits a different group of countries.

Sixth, we applied the synthetic control method to assess country performance on key outcomes after Gavi transition. As such, this study cannot be interpreted to provide evidence of causal inference of Gavi assistance on post-transition outcomes. Moreover, the synthetic control method does not assess nor directly control for factors that explain outcomes after transition, such as transition challenges identified in the literature, the number of vaccines, or the time a country has received Gavi support. Such factors could potentially be quantitatively evaluated using other econometric methods. However, only 8 units met the inclusion criteria for exposure to transition, which would severely constrain any such analysis.

Finally, this analysis is limited to 3 outcome variables. We considered other potential outcomes, such as pneumococcal conjugate vaccine immunization coverage among children aged 1 year (%); however, missing data would have introduced other constraints.

## CONCLUSIONS

This quasi-experimental study assessed post-Gavi country performance by comparing the observed vaccination coverage and post-neonatal mortality with the expected outcomes if the graduated countries had received continued Gavi support. Using the synthetic control method, we compared the outcomes of the graduating countries to the donor pool of countries continuing to receive Gavi support. We quantitatively tracked and reproduced the respective outcome trajectories of the transitioned countries to estimate the counterfactual. Overall, the results suggest that most countries have successfully transitioned from Gavi support by maintaining or further improving key outcomes compared to the estimated performance if Gavi assistance had continued. However, there are important heterogeneities, as previously discussed.

We argue that the coverage heterogeneities reflect the differences in contextual externalities as well as institutional strength and capacity. We found observational evidence that the transition from Gavi was more successful in countries that enjoyed a relatively stronger institutional environment earmarked by greater political stability and more effective government administrations. In addition, Gavi support entailed some level of external oversight, reporting, and accountability. These measures could have mitigated the impact of negative externalities, such as in the cases of Bosnia and Herzegovina and Ukraine. The findings suggest that improved immunization program performance achieved with assistance from Gavi could be sustained after transition.

Undoubtedly, the successful transition from external assistance is extremely complex and challenging, with many externalities. There is an increasing recognition that neither a country’s development nor the assistance it needs to progress is linear. This is particularly relevant in the context of fragile settings, shocks, and stressors, which are not mutually exclusive.^61^ In addition, country actors can leverage post-transition technical assistance and establish accountability mechanisms. For example, building on the Gavi-funded peer-to-peer learning platform for transitioned countries, the Linked Immunisation Action Network of technical experts from post-transition countries could form an advisory group that completes annual results reviews and provides technical assistance using identified challenges, lessons learned, and best practices to strengthen mutual accountability. Gavi and other stakeholders could provide initial financing to catalyze such activities, and out-year funding could be generated from membership dues and/or the private sector such as pharmaceutical companies. Finally, we recommend that Gavi systematize post-transition assessments and evaluations that leverage the expertise and experience of countries that no longer receive Gavi support to further encourage cross-learning.

## Supplementary Material

GHSP-D-22-00536-supplements.pdf

## Notes

**Peer Reviewed**

**First published online:** August 17, 2023.
