# Supplementary material for: Evaluating Country Performance After Transitioning From Gavi Assistance: An Applied Synthetic Control Analysis
Source: Glob Health Sci Pract. 2023 Aug 28;11(4):e2200536. doi: 10.9745/GHSP-D-22-00536 (PMC10461703; doi:10.9745/GHSP-D-22-00536)

Supplement to: Kolesar RJ, Spruk R, Tsheten T. Evaluating country performance after transitioning from Gavi assistance: an applied synthetic control analysis. *Glob Health Sci Pract*. 2023;11(4):e2200536. https://doi.org/10.9745/GHSP-D-22-00536

Supplement 1. Overview of GAVI Cofinancing Policies[1–3]

Policy Years	Country classifications	Cofinancing requirements	Basis of Determination
2008-2011	Fragile/ post conflict	charged $0.10 per dose for the first vaccine and $0.15 for subsequent vaccines	GNI per capita gross, United Nations' classification of least-developed countries, and GAVI's own determination of fragile or post-conflict status.
	Poorest	charged $0.20 per dose for the first vaccine and $0.15 for subsequent vaccines	
	Intermediate	charged $0.30 per dose for the first vaccine and $0.15 for subsequent vaccines	
	Least poor	charged amounts the same as for the "intermediate" group, but amounts increase by 15% per year*	
2012-2015	Low-income	charged $0.20 per dose for all vaccines	Informed by fiscal space analysis to determine levels of co-financing could reasonably be required of eligible countries at these different income levels. Which countries are likely to find it most difficult to meet their obligations to contribute to vaccine costs? And will graduating countries be able to fully absorb into their health budgets the cost of vaccines previously supported by the Gavi Alliance to maintain immunization gains.
	Intermediate	charged $0.20 per dose for all vaccines or the amount they were already paying upon entering the group, whichever is higher, and will face a 15% annual increase thereafter	
	Graduating	four-year ramp-up so that countries bear full cost when all GAVI support ends	
2015-present	Low-income-Initial	$0.20 per dose (no annual increase)	Primarily GNI per capita with adjustment for annual inflation.
	Phase 1-Preparatory transition	Following a three-year grace period, a starting year price fraction is multiplied by the weighted average Gavi price which is applied to individual vaccines; thereafter the price fraction is applied to all vaccines and country co-financing share increases by 15% per year.	
	Phase 2-Accelerated transition	Following the 'grace year', country co-financing share increases by 15% as it would have in Phase 1; subsequently, co-financing requirements increase linearly to reach 100% of the projected weighted average Gavi price used by the country in the first year without Gavi support. The timeframe for this increase was increased to 8years in accordance with the Eligibility and Transition Policy 4.0.	

*Because of its large population, India does not have to cofinance its GAVI vaccines and is excluded.

References

1. Gavi, the Vaccine Alliance. *Eligibility and Transition Policy Version 3.0*. Gavi; 2015. https://www.gavi.org/sites/default/files/document/gavi-eligibility-and-transition-policypdf.pdf
2. Gavi, the Vaccine Alliance. *Gavi Alliance Eligibility and Transition Policy Version 4.0*. Gavi; 2022. Accessed July 31, 2023. https://www.gavi.org/sites/default/files/programmes-impact/gavi-eligibility-and-transition-policy.pdf
3. Saxenian H, Cornejo S, Thorien K, Hecht R, Schwalbe N. An analysis of how the GAVI alliance and low- and middle-income countries can share costs of new vaccines. *Health Aff*. 2011;30(6):1122–1133. CrossRef. Medline

Supplement to: Kolesar RJ, Spruk R, Tsheten T. Evaluating country performance after transitioning from Gavi assistance: an applied synthetic control analysis. *Glob Health Sci Pract*. 2023;11(4):e2200536.
https://doi.org/10.9745/GHSP-D-22-00536

Supplement 2. Technical Methodological Description

Suppose $J+1$ countries are observed over $t = 1,2, \dots T$ periods with a single one being the treated country exposed to the end of Gavi assistance, and a sample of countries in the control group being unexposed to the treatment. Suppose that Gavi transition occurs at period $T_0 + 1$ where $1 < T_0 < T$. Let $Y_{i,t}^N$ denote the outcome variable of i-th country at time t in the absence of Gavi support; and, let $Y_{i,t}^I$ denote the same variable of i-th country at time t in the presence of Gavi support. Furthermore, we assume that the underlying institutional transition has no effect on the outcomes in the pre-intervention period which implies that $Y_{i,t}^N = Y_{i,t}^I$ for all i and $t < T_0 + 1$. Our aim is to estimate changes in the key outcomes following the end of Gavi support. By letting $\boldsymbol{\alpha} = \left(\alpha_1, \alpha_{T_0+1}, \dots \alpha_{1,T}\right)$ denote the vector of post-treatment effect where $t > T_0$, the effect of interest can be written as:

$$\alpha_{1,t} = Y_{i,t}^I - Y_{i,t}^N = Y_{1,t} - Y_{1,t}^N \quad (1)$$

where $Y_{1,t}$ denotes the outcome, and $Y_{1,t}^N$ is its counterfactual representation in the hypothetical absence of Gavi support. Since the counterfactual outcome trajectories are unobserved to the econometrician, it needs to be approximated and empirically recovered from reliable and observable pre-transition outcome characteristics. Therefore, we assume that $Y_{1,t}^N$ follows a latent factor model for each $i = 1,2, \dots N$ of the following form:

$$Y_{i,t}^N = \delta_t + \mathbf{X}_{i,t}'\theta_t + \lambda_t\mu_i + \epsilon_{i,t} \quad (2)$$

where δ_t is an unobserved factor common across all countries, $\mathbf{X}_{i,t}' \in \mathbb{R}^r$ is a vector of observed covariates unaffected by the transition, $\theta_t \in \mathbb{R}^r$ is a vector of parameters to be estimated, $\lambda_t \in \mathbb{R}^r$ is the vector of common unobserved factors, and $\mu_i \in \mathbb{R}^r$ is a vector of unknown factor loadings. The transitory shocks are assumed to be i.i.d distributed, and are denoted as $\epsilon_{i,t}$. One potential caveat against the latent model arises from the fact that the factor count is assumed fixed over time which disallows any presence of the structural break. To mitigate this caveat, we estimate the synthetic counterfactual trajectories by reweighing the control group so that an artificial (i.e. synthetic) control group for each treated country matches its actual counterpart on, and pre-transition outcome dynamics.

To construct the synthetic counterfactual outcome trajectories, let $W = \left(w_2, \dots, w_{J+1}\right)$ be a vector of weights with $w_j \geq \forall j$ where each value of Wrepresents a potential candidate for synthetic control group. For a given W, the outcome of the synthetic control group at time t is:

$$Y_{W,t} = \sum_{j=2}^{J+1} w_j Y_{j,t} = \delta_t + \theta_t\left(\sum_{j=2}^{J+1} w_j X_j\right) + \lambda\left(\sum_{j=2}^{J+1} w_j \mu_j\right) + \left(\sum_{j=2}^{J+1} w_j \epsilon_j\right) \quad (3)$$

Supplement to: Kolesar RJ, Spruk R, Tsheten T. Evaluating country performance after transitioning from Gavi assistance: an applied synthetic control analysis. *Glob Health Sci Pract*. 2023;11(4):e2200536. https://doi.org/10.9745/GHSP-D-22-00536

where $\exists W^*$ is such that the synthetic control group is set to match the transitioned country in pre-T_0 period so that we have $\sum_{j=2}^{J+1} w_j^* Y_{j,t} = Y_{1,t} \; \forall t \in \{1, \dots, T_0\}$ and $\sum_{j=2}^{J+1} w_j^* X_j = X_1$. Under these conditions, the synthetic control group constructed from W^* replicates the missing counterfactual scenario. By adopting the single-treatment synthetic control framework for one unit by Abadie et. al.,[1,2] an approximately unbiased estimator of $\alpha_{1,t}$ is given by:

$$\hat{\alpha}_{1,t} = Y_{1,t} - \sum_{j=2}^{J+1} w_j^* Y_{j,t} = Y_{1,t} - Y_{W^*,t} \quad (4)$$

where we perform a nested optimization and adopt constrained quadratic programming algorithm using an interior point method to solve the weight optimization problem.[3] This is performed by using the maximum likelihood optimizing technique, which yields the best-fitting weights from the regression matrix

$$\left\|\mathbf{X}_{j\in\{treated\},t<T_0} - \mathbf{X}_{0,t<T_0}\mathbf{W}\right\|_{\mathbf{V}} =$$

$$\sqrt{\left(\mathbf{X}_{j\in\{treated\},t<T_0} - \mathbf{X}_{0,t<T_0}\mathbf{W}\right)'\mathbf{V}\left(\mathbf{X}_{j\in\{treated\},t<T_0} - \mathbf{X}_{0,t<T_0}\mathbf{W}\right)}$$

where the nested optimization route allows us to seek highly similar combinations of countries that lie inside the convex hull of the treated countries' pre-transition **X** values. This ensures that pre-transition prediction error is minimized since all possible combinations of diagonal positive semidefinite matrices, and sets of weights for the best-performing convex combination of synthetic control countries. Among the control parameters for the nested optimization route, we follow the standard practices and set the violation tolerance constraint at 5% in a cross-sampling scheme that involves 1,000 iterations with 10% bound for the balancing covariates. Compared to the traditional difference-in-differences setup, our approach imposes much less restrictive assumptions on the consistency and unbiasedness of our estimates and permits a more flexible way of constructing a synthetic control group where pre-transition trends can be matched sufficiently well to compute the counterfactual scenario in response to the uprising.

The effects of the transition from Gavi support hinge on the statistical significance of the estimated counterfactual scenario. We compute the statistical significance of the counterfactual public health outcomes in the hypothetical absence of the phased transition by estimating the same outcome-linked model specification on each unaffected country, and obtain the distribution of placebo effects by iteratively shifting the treated country in the donor pool. The intuition behind the statistical significance of the public health and vaccination gap is simple. If the distribution of placebo effects yields many effects as large as the baseline set for the treated countries, then the estimated impact of the transition is most likely observed by chance alone.

Such test is based on the permutation of the phased transition to all unaffected countries, and does not impose any distribution on the random error term.[4] Assuming the effect of the transition

in the full post-treatment period is described by $\hat{\alpha}_{1,t}$, and let $\hat{\alpha}_{1,t}^{Placebo} = \{\hat{\alpha}_{j,t}: j \neq 1\}$ denote the distribution of in-space placebo effects. We compute two-tailed p-value for the underlying effect as follows:

$$\mathbb{P}_{t>T_0} = \Pr\left(\left|\hat{\alpha}_{1,t}^{Placebo}\right| \geq \left|\hat{\alpha}_{1,t}\right|\right) = \frac{\sum_{j\neq 1} 1 \cdot |\hat{\alpha}_{j,t}| \geq |\hat{\alpha}_{1,t}|}{J} \quad (5)$$

whereas for strictly positive effects, one-tailed p-value would be $\mathbb{P}_{t>T_0} = \Pr\left(\hat{\alpha}_{1,t}^{Placebo} \geq \hat{\alpha}_{1,t}\right)$. Notice that the treatment-related transition is not randomly distributed across the sample which implies that the placebo distribution serves a plausible randomization inference. But since the transition is not randomly assigned across the whole sample and thus does not satisfy the strict exogeneity condition, the underlying interpretation of the obtained probabilities invokes the proportion of countries that have an estimated effect of the assigned effect of the transition from Gavi at least as large as that of the treated countries. We further tackle the effect of the transition by using the post-transition RMSPE for the full set of treated countries, and compare it to the corresponding placebo counterpart for every unaffected country, and the derive the non-parametric p-values and their distributions to evaluate the uncertainty and significance of our estimates.[5–7] The key advantage of such approach is that it minimizes pre-program imbalances, enables the calculation of placebos based on large-scale treatment permutation, and builds the distributions of test statistics to assess whether the end of Gavi's financing produced a sizeable shift in the distribution of the effect, apart from making the effect on its own.

References

1. Abadie A, Diamond A, Hainmueller J. Synthetic control methods for comparative case studies: estimating the effect of California's tobacco control program. *J Am Stat Assoc*. 2010;105(490):493–505. CrossRef

2. Abadie A, Diamond A, Hainmueller J. Comparative politics and the synthetic control method. *Am J Pol Sci*. 2015;59(2):495–510. CrossRef

3. Vanderbei RJ, Shanno DF. An interior-point algorithm for nonconvex nonlinear programming. *Comput Optim Appl*. 1999;13(1/3):231–252. CrossRef

4. Galiani S, Quistorff B. The synth_runner package: utilities to automate synthetic control estimation using synth. *Stata J*. 2017;17(4):834–849. CrossRef
5. Abadie A, L'Hour J. A penalized synthetic control estimator for disaggregated data. *J Am Stat Assoc*. 2021;116(536):1817–1834. CrossRef
6. Wiltshire JC. allsynth: (Stacked) Synthetic Control Bias-Correction Utilities for Stata. 2022. https://justinwiltshire.com/allsynth-stacked-synthetic-control-biascorrection-utilities-for-stata
7. Ben-Michael E, Feller A, Rothstein J. The augmented synthetic control method. *J Am Stat Assoc*. 2021;116(536):1789–1803. CrossRef

Supplement to: Kolesar RJ, Spruk R, Tsheten T. Evaluating country performance after transitioning from Gavi assistance: an applied synthetic control analysis. *Glob Health Sci Pract*. 2023;11(4):e2200536. https://doi.org/10.9745/GHSP-D-22-00536

Supplement 3. Technical Results Description

Figure 2 shows the observed trajectories and synthetic counterparts of each country's DPT3 coverage from 2000-2020 among the eight countries that transitioned from Gavi support. The results reveal heterogeneity in the DPT3 coverage trend following graduation from Gavi support. As noted by the World Bank, immunization coverage levels are the result of a health system's inputs and efforts, therefore they frequently vary from year to year in a given country.[1] This renders some variation between the treated countries and their synthetic peers in the pre-transition period. Despite the fluctuations in the coverage rate, the synthetic control estimator provides a reasonably good fit between the observed trajectories of graduated countries and their synthetic controls retaining Gavi support. The predictive discrepancy between the graduating countries and their respective synthetic controls is consistently less than 5% of the pre-treatment error margin. The results reveal heterogeneity in the DPT3 coverage trend following transition, corroborating the notion that the effect of transition is far from uniform. At the end of the sample period (i.e. 2020), Albania, Bhutan, Guyana, and Turkmenistan overperformed their synthetic controls with DPT3 coverage 2.2, 2.5, 11, and 3.4 percentage points higher, respectively. Most notably, China achieved (near) universal DPT3 coverage by 2010 and therein the post-transition period exhibits the characteristics of a rapid and sustained breakaway from its synthetic control. China's DPT3 coverage is about 8 percentage points higher compared to its synthetic control in 2020.

By contrast, the estimates also reveal a negative effect in Bosnia & Herzegovina and Ukraine. For the former, we find an 16-percentage point deterioration from graduation: from 88% coverage in 2011 to 72% in 2020. Synthetic Bosnia & Herzegovina maintains coverage above 85% over the post-graduation period (exempting 2013). In relation to Ukraine, we observe a rampant deterioration in coverage post-transition until 2015 followed by the rapid recovery until 2020 where Ukraine's DPT3 coverage lags its synthetic control by approximately 12 percentage points. Notably, synthetic Ukraine maintains coverage over 90% over the entire post-transition period.

Figure 2 also highlights that Bosnia & Herzegovina, Turkmenistan, and Ukraine had important DPT3 coverage instability or deterioration before support from Gavi ended. The pre-graduation (2003) drop in immunization coverage in Turkmenistan is attributable to insufficient vaccination stock at all levels throughout the entire year[2] which recovered in the following year. Ukraine's dramatic post-graduation vaccination coverage drop necessitated a rescaling of its y-axis in Figures 2 and 3.

Figure 3 illustrates the observed trajectories of each country's measles coverage and their respective synthetic control estimates. The results are very similar to those shown in Figure 2 and suggest five distinctive impact types. First, we observe a general trend of coverage deterioration in Albania following graduation. However, this trend is closely aligned with its synthetic control. Second, following graduation from Gavi support Bhutan and Georgia outperform their respective synthetic controls, with both experiencing a coverage deterioration (and alignment with their

synthetic controls) in 2020. Third, China and Turkmenistan consistently outperform their synthetic controls with sustained (near) universal coverage. China's measles vaccination coverage and synthetic China is approximately 6 percentage points following a trend that can be observed with a dramatic increase in observed coverage beginning in the first year without any Gavi assistance (2006). This is in stark contrast to all other graduated countries. And, Guyana maintained high coverage post-graduation, outperforming its synthetic control.

Finally, prior to graduation we observe unstable measles coverage in Bosnia & Herzegovina and decreasing coverage in Ukraine. In relation to the former, the observed data show a sustained deterioration of measles vaccination coverage following Gavi graduation. In the final year of Gavi support, measles coverage stood at 90%, like its synthetic control. By the end of the sample period in Bosnia & Herzegovina, the vaccination rate plummeted to 68%: lagging its synthetic counterpart by about 25 percentage points (95% confidence interval = -23.1, -26.9). In relation to Ukraine, the observed measles vaccination rate tends to decrease substantially in the first years following graduation until 2016, followed by a rapid and uninterrupted recovery up to the end-of-sample years. In quantitative terms, Ukraine's rate of vaccination against measles in 2020 is around 12 percentage points lower relative to its synthetic control (95% confidence interval = -5.5, -18.5). The gradual closure of the gap indicates a large improvement from 2016 when the gap between Ukraine and its synthetic control group stood at about 50 percentage points (95% confidence interval = -43.5, -56.4).

Figure 4 shows post-neonatal mortality rates and the respective synthetic control estimates for the eight countries. In contrast to immunization coverage indicators which frequently vary from year to year in a given country, child mortality responds to a number of determinants and change slowly.[1] Thus, the synthetic control estimator provides an excellent fit between the treated countries' trajectories and their synthetic control groups with almost zero imbalance therein. Three key insights can be drawn from our analysis of the respective mortality impact post-transition.

First, a discernible decrease in the mortality rate can only be expected in those countries having notably higher mortality rate relative to the benchmark levels. The results reveal a sustained drop in the post-neonatal mortality trajectory in Albania. The observed rates outperform the synthetic control with the end-of-sample mortality rate reduction approximately 3 per 1,000 live births lower than expected. By contrast, the trend decrease in post-neonatal mortality in Turkmenistan underperforms with reductions leveling off in 2012 and a difference of about 5 per 1,000 live births in 2020, compared to its synthetic control. In addition, we note that Albania, Bosnia & Herzegovina, China, Georgia, and Ukraine have very low post-neonatal mortality rates (i.e. less than 10 per 1,000) at transition, with near exact pre-transition matching among their respective synthetic counterparts. Since these countries already have relatively low mortality rates, the potential for additional improvement is limited. Finally, the post-transition non-discrepancy between the transitioned countries and their control groups among all other countries suggests that those countries have maintained progress in post-neonatal mortality rate reductions similar to what would have been expected if Gavi support had continued.

Supplement to: Kolesar RJ, Spruk R, Tsheten T. Evaluating country performance after transitioning from Gavi assistance: an applied synthetic control analysis. *Glob Health Sci Pract*. 2023;11(4):e2200536. https://doi.org/10.9745/GHSP-D-22-00536

Figure 5 reports the condensed composition of synthetic control groups, summarizing the frequency of non-zero weight for each donor country not affected by the treatment itself. A higher frequency indicates a stronger and more influential contribution of that donor country to the vaccination and mortality trajectories. This is because it most closely matches and reproduces the trajectories of the treated countries vaccination coverage and mortality paths in the period before Gavi assistance ended. The schematic composition of synthetic control groups unveils both notable contrasts and similarities across the three outcome-linked specifications. For instance, the most frequent non-zero contributing countries in the series of DPT3 specifications include Sri Lanka, Uzbekistan, Mongolia, and Rwanda whilst Central African Republic, Pakistan, and Kenya contribute the lowest degree of influence.

Furthermore, in the measles specification, Uzbekistan stands out as the most important donor with an large frequency ratio compared to the other donors. In addition, the composition of synthetic control groups in the mortality specifications highlights Moldova, Cuba, Armenia and Uzbekistan as the most influential donor countries affecting the composition of control groups opposed to Sri Lanka, India, and Indonesia which stand out as the least influential donors based on the collected weight share matrix. Overall, the compositional differences across the synthetic control groups emphasizes a reasonably good quality of the fit provided by the convex combinations of the implicit attributes of the donor countries in capturing and tracking the vaccination outcome trajectories of the graduating countries despite some differences in the mixture of countries best synthesizing pre-graduating outcome trajectories. It should be noted that the full set of treated countries has a reasonably good pre-graduating trajectories of vaccination coverage quality of the fit, and an excellent fit with the control group with respect to the post-neonatal mortality trajectories. For instance, China's pre-graduation DPT3 coverage trajectory is best reproduced as a convex combination of the implied attributes of Mongolia (42%), Indonesia (22%), Uzbekistan (11%), India (12%), Armenia (10%), Pakistan (3%), and Moldova (<1%), accordingly. In terms of additional example, Bosnia and Herzegovina's synthetic control group for pre-graduating DPT3 trajectory consists of the weighted combination of the outcome and auxiliary characteristics of Moldova (40%), Nicaragua (24%), Vietnam (21%), Djibouti (10%), Central African Republic (5%), and Cuba (<1%), respectively. Similar contrasts can be observed across the measles vaccination coverage and mortality-related specifications.

We compute the statistical significance of the counterfactual public health outcomes and obtain the distribution of placebo effects. The resultant p-values can be interpreted as "the probability of obtaining an estimate at least as large as the one obtained for the unit representing the case of interest when the intervention is reassigned at random in the data set."[3] Table 2 presents the key parameters from the placebo analysis. The results suggest that assigning the transition date at random to the countries that did not transition does not yield statistically significant estimates, providing reasonable plausibility of our estimates.

References

Supplement to: Kolesar RJ, Spruk R, Tsheten T. Evaluating country performance after transitioning from Gavi assistance: an applied synthetic control analysis. *Glob Health Sci Pract*. 2023;11(4):e2200536. https://doi.org/10.9745/GHSP-D-22-00536

1. Batson AE, Bos ER. *Using Immunization Coverage Rates for Monitoring Health Sector Performance: Measurement and Interpretation Issues*. World Bank Group; 2000. Accessed July 31, 2023. https://documents.worldbank.org/en/publication/documents-reports/documentdetail/607721468763783070/using-immunization-coverage-rates-for-monitoring-health-sector-performance-measurement-and-interpretation-issues
2. Government of Turkmenistan. *Progress Report to the Global Alliance for Vaccines and Immunization (GAVI) and The Vaccine Fund*. Gavi, the Vaccine Alliance; 2004. Accessed July 31, 2023. https://www.gavi.org/sites/default/files/document/annual-progress-report-turkmenistan-2003pdf.pdf
3. Abadie A, Diamond A, Hainmueller J. Comparative politics and the synthetic control method. *Am J Pol Sci*. 2015;59(2):495–510. CrossRef

Supplement to: Kolesar RJ, Spruk R, Tsheten T. Evaluating country performance after transitioning from Gavi assistance: an applied synthetic control analysis. *Glob Health Sci Pract*. 2023;11(4):e2200536. https://doi.org/10.9745/GHSP-D-22-00536

Supplement 4. In-space Placebo Sensitivity Analysis results

Figure A. Inference on the null effect of graduation from Gavi on DPT3 vaccine coverage across full treatment sample

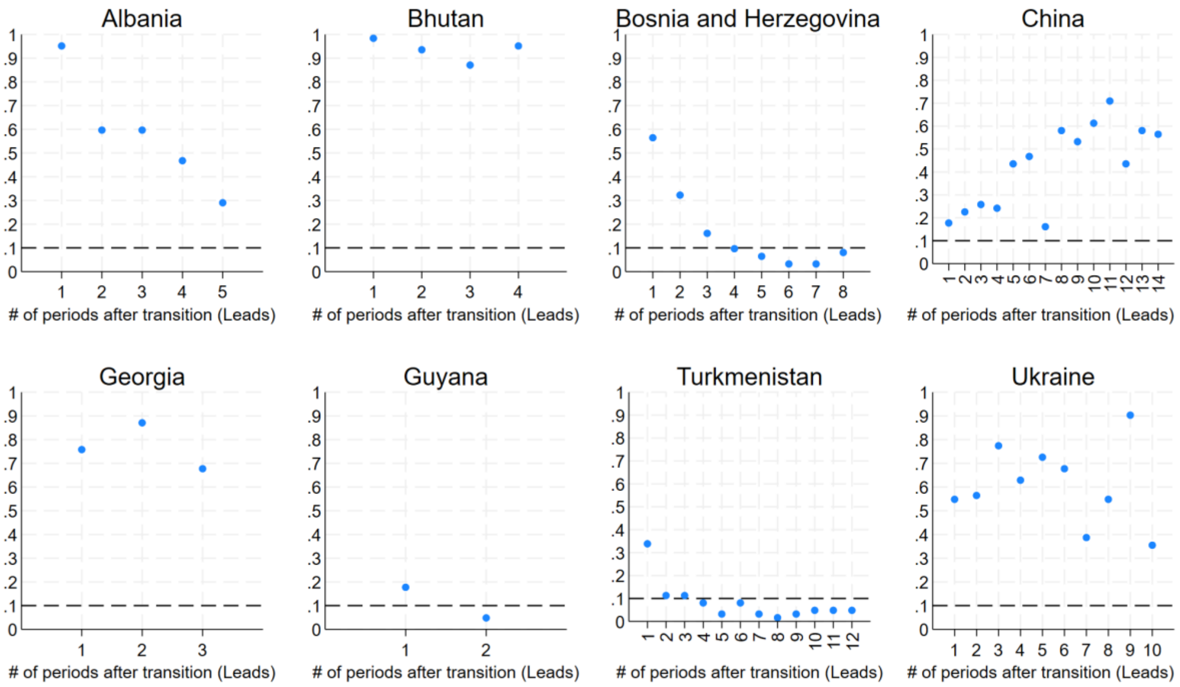

Figure B. Inference on the null effect of graduation from Gavi on measles vaccine coverage across full treatment sample

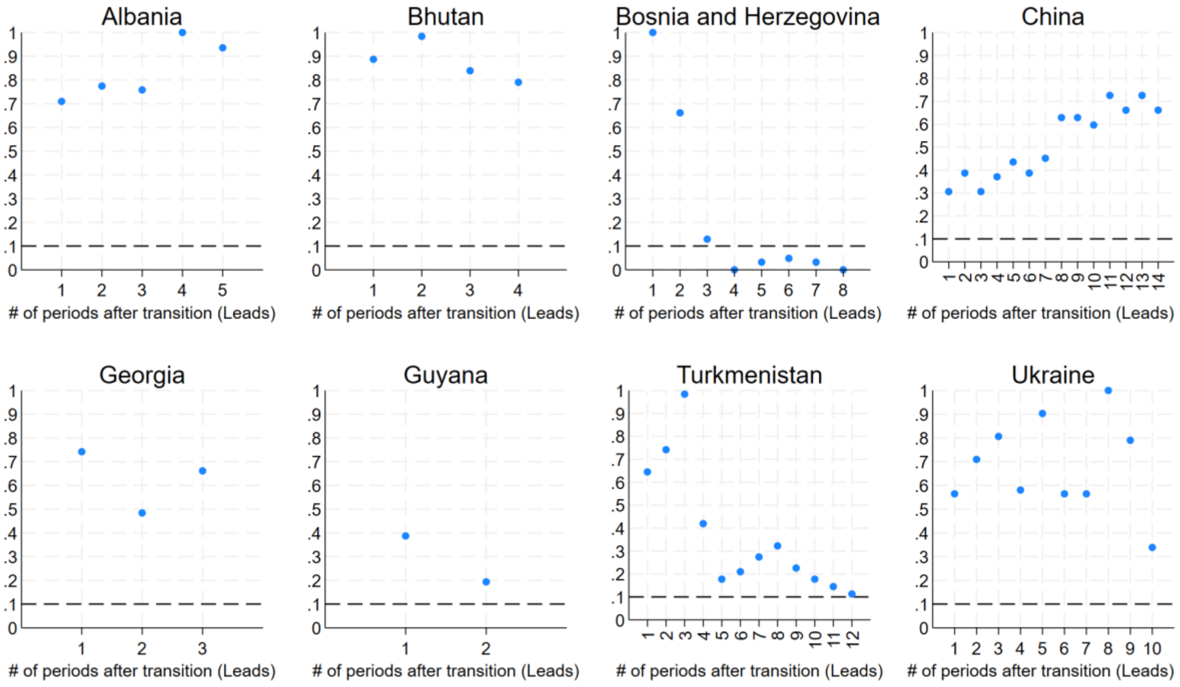

Supplement to: Kolesar RJ, Spruk R, Tsheten T. Evaluating country performance after transitioning from Gavi assistance: an applied synthetic control analysis. *Glob Health Sci Pract*. 2023;11(4):e2200536. https://doi.org/10.9745/GHSP-D-22-00536

Figure C. Inference on the null effect of graduation from Gavi on post-neonatal mortality across full treatment sample

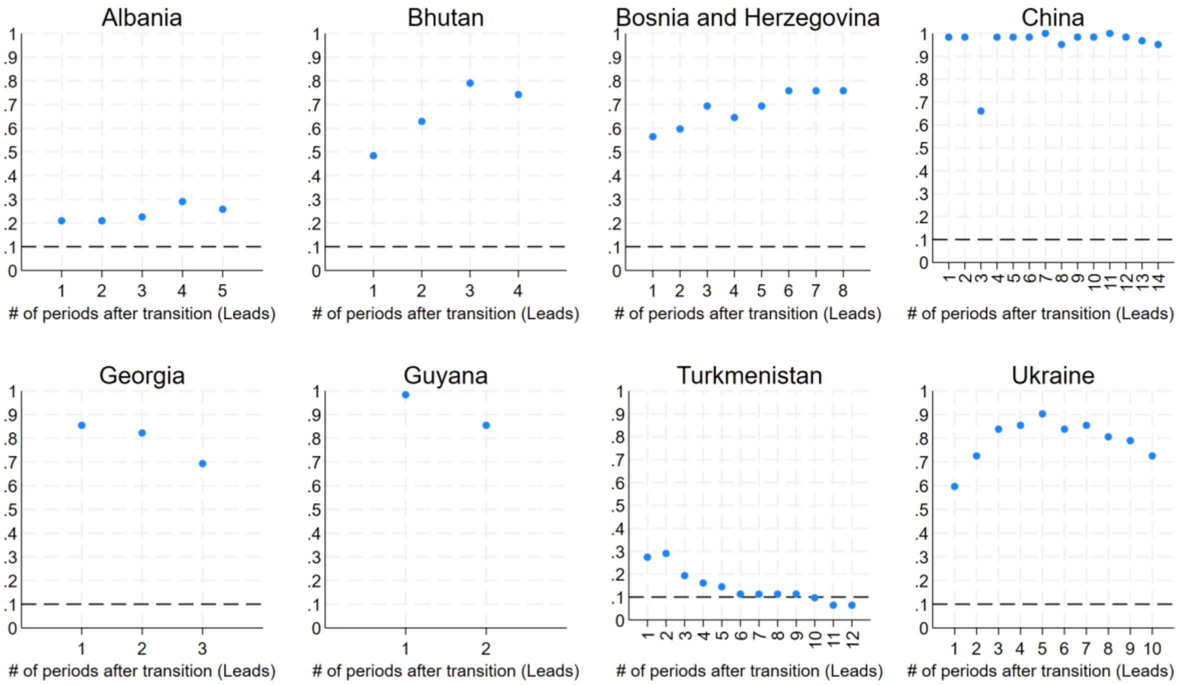

Supplement: GHSP-D-22-00536-supplements.pdf [file GHSP-D-22-00536-supplements.pdf]
